# Translocation of chlorantraniliprole and cyantraniliprole applied to corn as seed treatment and foliar spraying to control *Spodoptera frugiperda* (Lepidoptera: Noctuidae)

**DOI:** 10.1371/journal.pone.0229151

**Published:** 2020-04-01

**Authors:** Maiquel P. Pes, Adriano A. Melo, Regina S. Stacke, Renato Zanella, Clérison R. Perini, Fábio M. A. Silva, Jerson V. Carús Guedes

**Affiliations:** 1 Laboratory of Integrated Pest Management, Department of Crop Protection, Federal University of Santa Maria, Santa Maria, Rio Grande do Sul, Brazil; 2 Department of Chemistry, Federal University of Santa Maria, Santa Maria, Rio Grande do Sul, Brazil; 3 Latin America Technical Insecticide Manager, FMC Agricultural Solutions, Paulínia, São Paulo, Brazil; USDA-ARS Southeast Area, UNITED STATES

## Abstract

The translocation of chemical insecticides in corn plants could enhance the control of *Spodoptera frugiperda*, based on their application form. Chlorantraniliprole and cyantraniliprole were applied via seed treatment and foliar spray in corn (VE and V3) to characterize the systemic action of both molecules in leaves that appeared after application. Bioassays with *S*. *frugiperda* and chemical quantification in LC-MS/MS confirmed the absorption and upward translocation of chlorantraniliprole and cyantraniliprole by xylem to new leaves. Both insecticides caused the mortality of larvae up to stage V6 (57.5±9.5% for chlorantraniliprole and 40±8.1% for cyantraniliprole), indicating the translocation of insecticides into leaves of corn plants when applied via seed treatment. However, the translocation of chlorantraniliprole and cyantraniliprole from sprayed leaves to new leaves was not observed, regardless of the stage of application plus the next first, second and third stages. An increased dosage of cyantraniliprole did not influence on its translocation in plant tissues, however, it influenced on the present amount of active ingredient. The application of chlorantraniliprole and cyantraniliprole in seed treatment is an important alternative for integrated pest management. The absorption and redistribution capacity of chlorantraniliprole and cyantraniliprole throughout the plant confer a prolonged residual action with satisfactory control of *S*. *frugiperda*.

## Introduction

Insecticide translocation is crucial for insect pest control in plants, as it allows the insecticide to be homogeneously distributed, reaching plant organs and being used as a food source by the insect [1]. This feature may aid in the management of fall armyworm, *Spodoptera frugiperda* (J. E. Smith, 1797) (Lepidoptera: Noctuidae), in corn (*Zea mays* L.). *S*. *frugiperda* can decrease corn yields up to 57%, depending on the crop season and the hybrid [2], feeding inside the corn whorl, hindering its control by foliar spraying [3].

The low adoption of Integrated Pest Management (IPM) and Insect Resistance Management (IRM) in corn fields in Brazil has led to the rapid evolution of resistance of *S*. *frugiperda* to transgenic corn events expressing insecticidal proteins from *Bacillus thuringiensis* Berliner (*Bt*) [4]. Currently, in Brazil, the application of chemical insecticides has been necessarily adopted in *Bt* corn events that presented efficacy loss to resistant populations of fall armyworm [5]. Therefore, the use of systemic insecticides may play an important role in the management of this economically important pest of corn, as it was reported for diamides applied in the seed treatment of soybeans [6].

Insecticides of the diamides chemical group are widely used to control pests in many commercial crops worldwide [7]. Diamides was labeled for use over 10 years ago (2008) [8] and are classified in the group 28 by the Insecticide Resistance Action Committee (IRAC), acting as modulators of ryanodine receptors [9]. Since their commercialization, diamides have been widely used for pest management in various countries, reaching one billion USD in sales in 2016 [10]. Chlorantraniliprole and cyantraniliprole are anthranilic diamides that are currently labeled for use both as seed treatment and foliar application in Brazil [11]. Chlorantraniliprole was the first anthranilic diamide developed and marketed, with wide action on lepidopterans [12] and other chewing insects [10]. Cyantraniliprole is part of the second generation [13], with a broader action spectrum on lepidopterans, dipterans, coleopterans, hemipterans and thysanopterans [14].

Physicochemical characteristics, such as water solubility and Log Pow (octanol/water partition coeficient) [15], are important for the translocation of the active ingredient in the plant [16]. The physicochemical properties of chlorantraniliprole and cyantraniliprole allow the upward movement of both molecules through xylem, especially when applied via seed treatment or in the soil, near the root system [10]. Cyantraniliprole has lower Log Pow and a higher solubility in water, compared to chlorantraniliprole [13–17]. This feature provides a greater mobility of cyantraniliprole in the plant [10].

The upward movement of chlorantraniliprole and cyantraniliprole applied in seed treatment via xylem was demonstrated in soybean plants in the control of *S*. *frugiperda* in laboratory bioassays [6]. The effective control of pests, including *Ostrinia nubilalis* [18], *Delia platura* [18] and *Diatraea saccharalis* [19], was observed in field experiments with soil application and in seed treatment with chlorantraniliprole and cyantraniliprole. Translaminar and acropetal movements were also observed for cyantraniliprole applied as foliar spray [15]. The translocation of this insecticide can be influenced by several factors, namely: environmental conditions, physiological conditions, plant age, and plant species [20].

Corn is one of the most important crops worldwide, having the fall armyworm as a primary pest, which requires multiple control tactics, including the use of chemical insecticides such as diamides. Nonetheless, the translocation of anthranilic diamides in corn plants is little known and can be a field-relevant information to manage the fall armyworm. Therefore, the objective of this study was to characterize and to quantify the translocation of field recommended rates of insecticides chlorantraniliprole and cyantraniliprole in corn plants, applied via seed treatment and foliar spraying in different phenological stages to control *S*. *frugiperda*.

## Material and methods

### Corn plant growth

A non-Bt corn variety, P1921 (Pioneer, Brazil), was used in this study. Individual plants were cultivated in 5L polyethylene containers, filled with a mixture of 30% substrate (Mecplant) and 70% soil, kept in a greenhouse (photoperiod 14:8h; 25±2°C; 75±5% RH), until they reached the desired phenological stage (V1, V2, V3, V4, V5 and V6, respectively). Irrigation was performed daily to ensure sufficient moisture for plant growth.

### Larvae of *Spodoptera frugiperda*

Larvae of *S*. *frugiperda* used in the experiments were obtained from a rearing of the Laboratory of Integrated Pest Management (LabMIP-UFSM). The larvae were kept in the rearing room (photoperiod 16:8h; 25±20C; 75±5% RH) and fed an artificial diet [21]. Prior to infestation (96 h), the larvae were fed with untreated corn leaves for acclimatization.

### Application of treatments

#### Seed treatments

For the application in seed treatment (ST), chlorantraniliprole (Dermacor^®^, DuPont, USA) and cyantraniliprole (Fortenza^®^, Syngenta, Switzerland) were used. Chlorantraniliprole (dosage of 45g a.i. per 60,000 seeds) and cyantraniliprole (150g a.i. dosage per 100 kg of seed) were dosed with a micropipette and applied to corn seeds in plastic bags containing 500 grams of seeds. Soon after, the bags were shaken manually until the seeds presented uniform coverage. The seeds were kept for one hour in the shade, just to lose the seed treatment moisture, and then immediately sowed in the containers. Additionally, a control treatment was conducted with seeds free of insecticides.

#### Foliar spraying

The insecticides used for aerial spraying were chlorantraniliprole (Premio^®^, DuPont, USA) and cyantraniliprole (Benevia^®^, DuPont, USA). The foliar sprayings of chlorantraniliprole (dosage of 25 g a.i. ha^-1^) and cyantraniliprole (dosages of 50 g a.i. ha^-1^ and 150 g a.i. ha^-1^) were performed in stages VE and V3. The insecticides were applied in a spray chamber (Generation III Spray Booth, Minnesota, USA) equipped with fan-type tip (XR 110.01, Teejet, USA), with 30 psi of pressure, 3.6 km h^-1^ velocity and volume of 100 L ha^-1^. The containers with corn plants were covered with plastic bags to prevent chlorantraniliprole and cyantraniliprole from reaching the soil at the time of spraying and being absorbed by plant roots. After the application and drying of leaf surface, the plastic bags were removed, the plants were then returned to the greenhouse and kept under controlled conditions (photoperiod 14:8h; 25±2°C; 75±5% RH) during the assessments. Additionally, a control treatment was performed with plants that did not receive foliar spraying of insecticides.

### Bioassays with *Spodoptera frugiperda*

**Fig 1:**
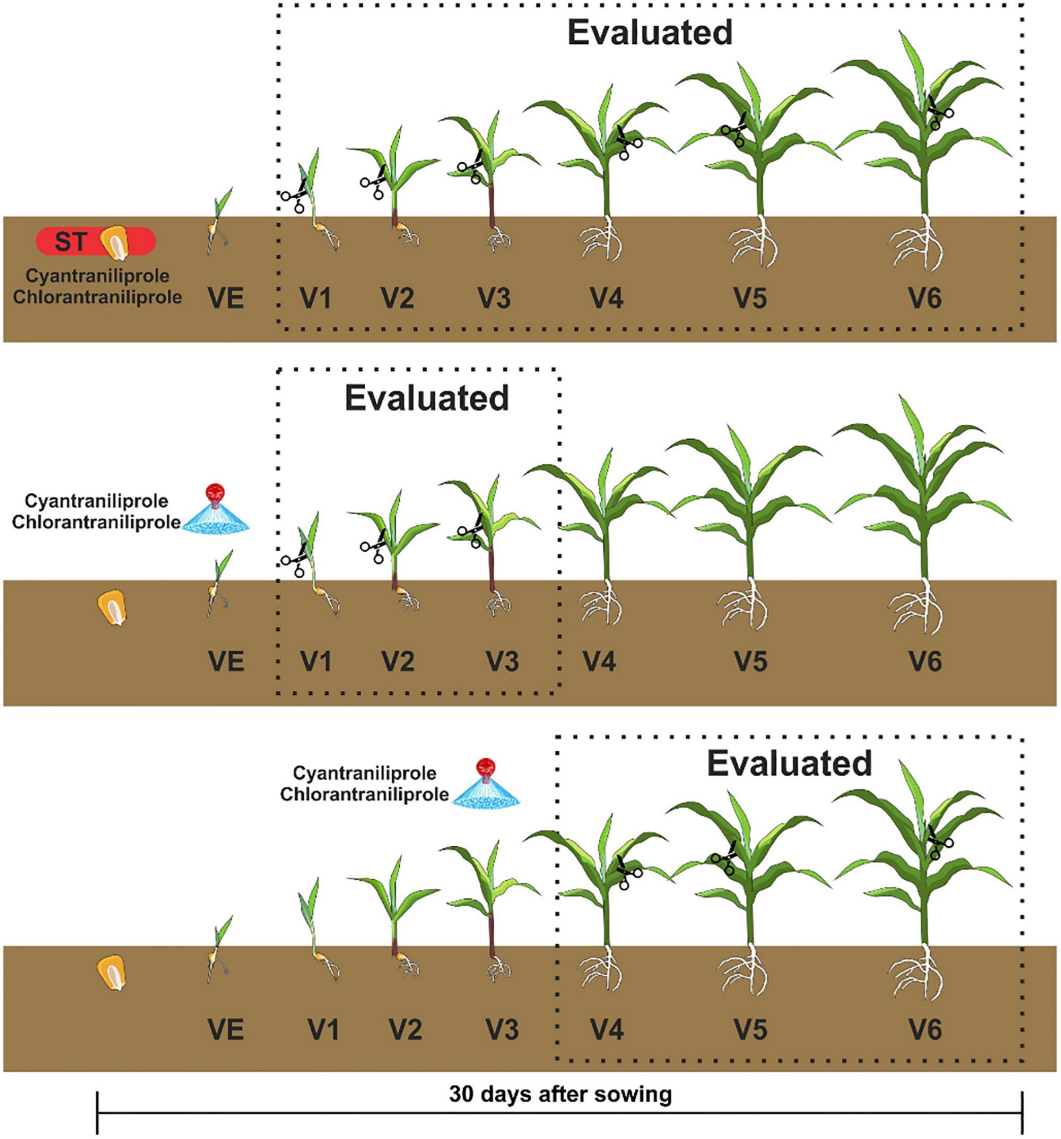
Application times and evaluation stages of chlorantraniliprole and cyantraniliprole insecticides in corn plants.

The larvae of *S*. *frugiperda* at L3 stage were used to feed on corn leaves that appeared after the application via seed treatment (ST) or foliar spraying (Fig 1). In the experiments with ST, the larvae were fed on leaves from stages V1 (9 days), V2 (12 days), V3 (16 days), V4 (20 days), V5 (25 days) and V6 (30 days). For foliar spraying, in stage VE, larvae were fed on leaves of stages V1, V2 and V3, and for spraying in V3, larvae were fed on leaves V4, V5 and V6. Fully expanded leaves were removed from the plants and supplied whole to the larvae until stage V3. For V4, V5 and V6 stages, due to their size, leaves were divided into three parts using scissors in the transverse direction: base, middle and apex of the leaf. Therefore, all leaf parts were contemplated.

For the bioassay experiments on seed treatments and foliar spraying were used four replicates with 10 corn plants (one plant in each container) per growth stage of evaluation. Considering all growth stages (V1, V2, V3, V4, V5 and V6) on the seed treatment experiment, a total of 240 plants per insecticide treatment were used. Therefore, a total of 480 plants were used. For the experiment with foliar spraying at VE, 120 corn plants per insecticide treatment were used, totalizing 360 plants. The same number of plants were used for the experiment with foliar spray at V3. The control treatment was also performed with four replicates and 40 plants per growth stage, totalizing 240 plants. In this sense, a total of 1440 plants were used in the bioassays, allowing to collect only one leaf per plant to feed the larvae, in order not to impair the insecticide movement in plant tissues.

The leaves were placed individually in polyethylene containers of 100ml, containing a 2-mm layer of 2.5% water-carrageenan mixture to keep the turgidity of the leaf tissue during evaluation. Corn leaves were separated from the water-carrageenan mixture by a filter paper disc. Subsequently, each container received a larva of *S*. *frugiperda* in L3 stage. Each treatment consisted of four replicates, each replicate consisted of 10 larvae distributed individually in each container, totaling 40 larvae per treatment. The containers were kept in a room with controlled environment (photoperiod 14:10h; 26±2°C; 75±5% RH). Dead larvae were counted four days after infestation.

### Sampling of corn leaves for LC-MS/MS

To quantify insecticides applied via ST and via foliar spraying, the exact leaves that determine the stages V1, V2, V3, V4, V5 and V6 (Fig 1) of corn plants were collected individually from 20 plants (5 leaves per replicate on each stage). All leaves of each replicate (from 510 containers) were placed individually in plastic bags and stored in an Ultrafreezer (Model U360) at -80°C.

For the leaf sampling in the experiment of seed treatments, 20 plants were used per each growth stage (V1-V6), totalizing 120 per insecticide treatment. On foliar spraying at VE and V3, the leaves were sampled over 120 plants per insecticide treatment, comprising a total of 360 plants over V1-V6 stages. Additionally, the control treatment was performed with 20 plants per growth stage, with a total of 120 plants. Therefore, 720 corn plants were used to quantify chlorantraniliprole and cyantraniliprole in LC-MS/M without disturbance on insecticide translocation.

### Samples preparation

The leaves from each replicate were lyophilized (Lyophilizer SL-404/B) for 24 h until the complete removal of water. The leaves were ground in a knife mill (Model SKU: SP227-01) for 1 min, until a fine powder was obtained. For extraction, the QuEChERS original method with modifications was used, in which 0.3g of sample was weighed on a precision scale and placed in a Falcon tube (CRAL—Articles for Laboratory Ltda.). In each Falcon tube containing a leaf sample, we added 1 mL of acetonitrile and a package of the original QuEChERS Kit (Bond Elut—Agilent Technologies) [22]. The sample was shaken for 1 min in vortex and then centrifuged (Eppendorf 5810 R centrifuge) for 3 min at 3400 rpm. After this, we collected 1ml of the supernatant solution, which was shaken in vortex for 30.0 seconds along with 0.3g of MgSO_4_ (Synth), 0.05g of primary secondary amine (PSA) (Agilent Technologies), and 0.004g of graphite carbon (Sigma-Aldrich). The samples were centrifuged again for 3 min at 3400 rpm and then 1 mL of the supernatant was removed and filtered in Aura MT Syringe Filter, PTFE, Hydrophilic, 0.45 Pore Size, 25 mm Diameter and taken for chromatographic analysis.

### Quantification of chlorantraniliprole and cyantraniliprole in LC-MS/MS

Chlorantraniliprole and cyantraniliprole were quantified analytically using a liquid chromatography system coupled to mass spectrometry in tandem mode (LC-MS/MS), model Varian 320-MS (Walnut Creek, USA). The equipment consisted of a 212-LC quaternary pump, column oven with a degassing system, ProStar 410 automatic sampler and a triple detector MS 320-MS quadrupole (TQ) with API source, using electrospray ionization mode. Software Varian Workstation 6.9.2 (Walnut Creek, USA) was used to collect the data. The chromatographic separation was performed in a column (Pursuit XRS Ultra C18 (100 x 2 mm, 2.8 μm particle size) of Agilent (Santa Clara, USA). The column was kept at 30°C with an injection volume of 10 μL. The mobile phase was an aqueous solution of formic acid 0.1% and acetonitrile 0.1% of formic acid in isocratic elution mode with flow rate 0.15 mL.min^-1^. The conditions used in the detector were: ionization source temperature 150°C; desolvation temperature 250°C; nebulizing gas pressure (N_2_) at 20 psi; pressure of desolvation gas (N_2_) at 40 psi; and collision gas pressure (Ar) 1.8 mTorr. The active ingredients were analyzed using positive ionization mode (ESI+), generating molecular ions [M + H]+ with m/z 483.9 for chlorantraniliprole and 475 for cyantraniliprole. For chlorantraniliprole, the ion fragments 483.9 > 286 and 483.9 > 452.5 were used for quantification and confirmation, respectively. For cyantraniliprole, the ion fragment 475 > 285.7 was used for quantification and 475 > 443.8 for confirmation. For quantification, analytical curves were prepared with standard solutions for each active ingredient within the linear range (r^2^ ≥ 0.99), and by interpolation of peaks integration referring to the analytes of choice in the curves, the result was expressed in mg. kg^-1^.

The limits of detection (LOD) and quantification (LOQ) of the active ingredients were estimated using the signal-to-noise ratio method. The LOD was defined as the lowest concentration that the analytical signal could be reliably differentiated with a 3:1 signal-to-noise ratio. The LOQ was determined as the lowest peak concentration that produced a 10:1 signal-to-noise ratio [23]. For chlorantraniliprole, the corresponding values were LOD: 0.075 mg a.i. kg^-1^ and LOQ: 0.250 mg a.i. kg^-1^. The corresponding values for cyantraniliprole were LOD: 0.150 mg a.i. kg^-1^ and LOQ: 0.500 mg a.i. kg^-1^.

### Experiment design and statistical analysis

The experimental design was completely randomized to evaluate insect mortality and for quantification of anthranilic diamides. The mortality of fall armyworm was corrected based on the Abbott’s formula [24] and then the data was submitted to ANOVA analysis. The chemical quantification data were analyzed separately for each active ingredient as a bifactorial, comparing application forms with corn leaves. The means of mortality and chemical quantification were compared by the Tukey test (*P* = 0.01) with Sisvar^®^ software version 5.6 [25]. Additionally, a Pearson correlation was performed between larvae mortality (%) and chemical quantification for each insecticide treatment.

## Results

### Bioassays on *Spodoptera frugiperda*

**Fig 2:**
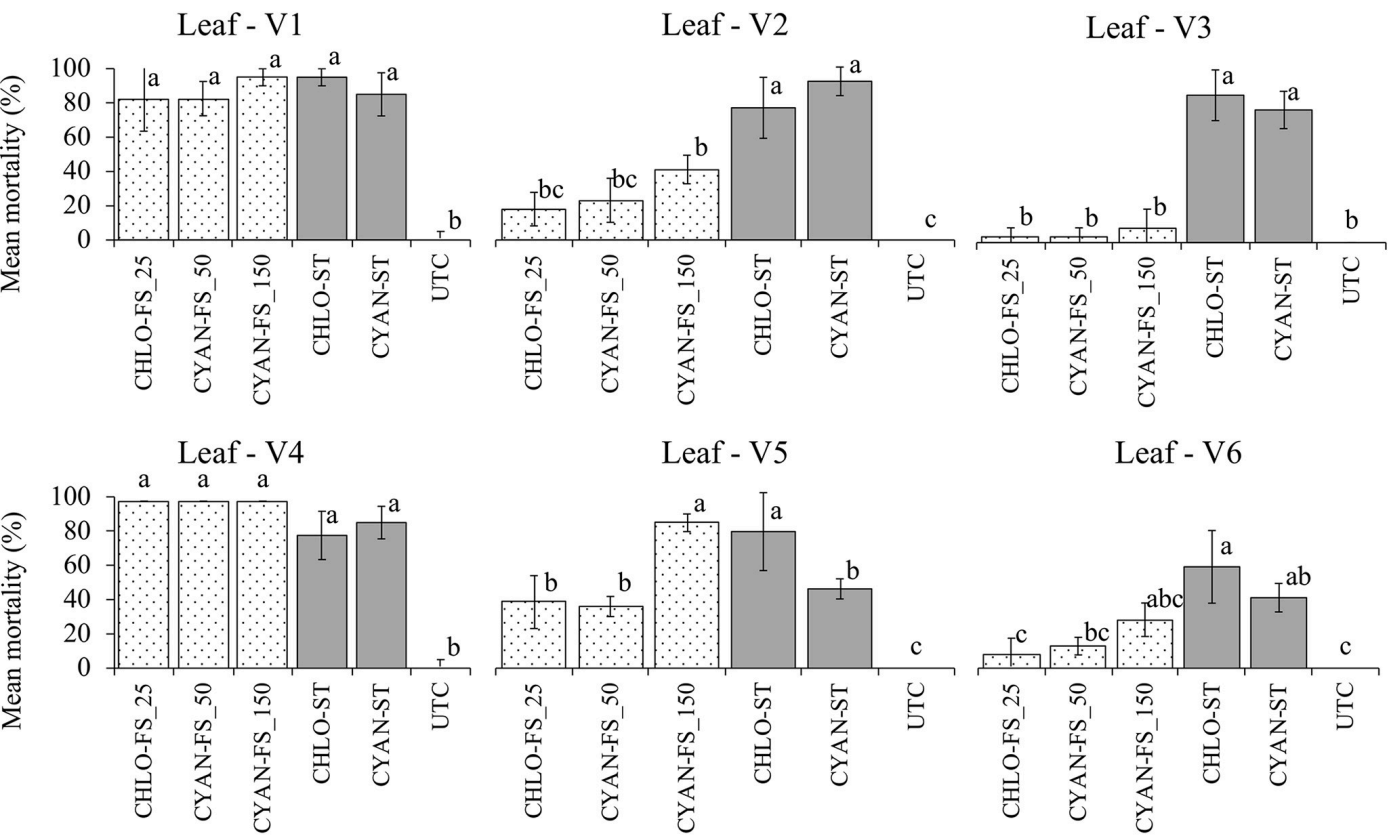
Mortality of *Spodoptera frugiperda* due to application of insecticides chlorantraniliprole and cyantraniliprole via seed treatment and foliar spraying in stages VE and V3 in corn. Means with the same letter do not differ significantly by the Tukey test *P* = 0.01. In foliar spray are CHLO-FS_25: chlorantraniliprole at 25 g ai; CYAN-FS_50: cyantraniliprole at 50 g ai; CYAN-FS_150: cyantraniliprole at 150 g ai; and in seed treatment are CHLO—ST: chlorantraniliprole; CYAN-ST: cyantraniliprole—ST; UTC: untreated. ANOVA: V1 (F = 40.744; df = 5; P<0.01); V2 (F = 53.845; df = 5; P<0.01); V3 (F = 74.950; df = 5; P<0.01); V4 (F = 106.469; df = 5; P<0.01); V5 (F = 29.594; df = 5; P<0.01); V6 (F = 10.268; df = 5; P<0.01).

The treatments with insecticides showed differences in the mortality of *S*. *frugiperda* when applied in seed treatment (ST) and foliar spraying (VE and V3), with a difference in concentration of active ingredients in the leaves, according to the type of application (Fig 2). For insecticides applied as ST, the highest mortality was accomplished with leaves in V1 growth stage, with 95±5% for chlorantraniliprole and 85±12.6% for cyantraniliprole. Chlorantraniliprole and cyantraniliprole applied via ST showed significant larvae mortality until stage V6 of corn plants, with 57.5±9.5% and 40±8.1%, respectively (Fig 2). These results demonstrate the translocation of insecticides from the seed treatment to all leaves evaluated and show the long residual effect of anthranilic diamides to control *S*. *frugiperda*, when applied as ST.

All treatments with insecticides applied via foliar spraying in VE resulted in a high mortality of *S*. *frugiperda* only for leaf V1, ranging from 82.5±19% to 95±5% (Fig 2). On the contrary, for the leaves at V2 and V3 stages, the application of chlorantraniliprole (25 g a.i. ha^-1^) and both dosages of cyantraniliprole (50 and 150 g a.i. ha^-1^) resulted in a lower mortality of larvae (ranging from 40±8.1% to 2.5±5%). The results of leaf V5 after spraying at V3 growth stage showed that the highest dosage of cyantraniliprole (150 g a.i./ha^-1^) applied via foliar spraying resulted in a significant larvae mortality of 82.5±5%, compared to cyantraniliprole (50 g a.i. ha^-1^) with 35±5.7% of mortality.

Spraying at VE with leaves collected in V3 and spraying at V3 with leaves collected in V6, showed no significant difference in larvae mortality, compared to control treatment, ranging from 2.5±5% to 27.5±9.5% of mortality (Fig 2). These results demonstrate that chlorantraniliprole and cyantraniliprole were possibly present at low concentrations in the leaf tissues, or that they did not translocate to untreated leaves.

### Quantification of chlorantraniliprole and cyantraniliprole in leaves

There was significant interaction for chlorantraniliprole and cyantraniliprole between the application form and leaves collected in the different stages (*P* < 0.01) (S1 Table). Data on foliar application of chlorantraniliprole (25 g a.i. ha^-1^) in stage VE show that the active ingredient concentration reduced over time, resulting in non-detection of the product in leaves of stage V3. The same occurred when chlorantraniliprole (25 g a.i. ha^-1^) was applied by foliar spraying in stage V3 and the product was not detected in the leaf V6. However, when chlorantraniliprole was applied via seed treatment at field rate of 45 g a.i. ha^-1^, all leaves showed product concentration, confirming the absorption and translocation capacity of this active ingredient to new leaves when applied via ST (Table 1).

**Table 1:** Foliar concentration of active ingredient chlorantraniliprole applied in foliar spraying and seed treatment for different leaves of corn plants.

	Treatment	Dosage (g a.i.)	Application type	Leaves (mg a.i./kg^-1^)					
				**V1**		**V2**		**V3**	
**VE X ST**	Chlorantraniliprole	25	Foliar^c^	0.46 ± 0.08	^a^bA	0.43 ± 0.06	aA^b^	0.0 ± 0.0	aB
	Chlorantraniliprole	45	ST^d^	0.69 ± 0.04	aA	0.37 ± 0.07	aB	0.07 ± 0.0	aC
CV (%)	14.60								
				**V4**		**V5**		**V6**	
**V3 X ST**	Chlorantraniliprole	25	Foliar	6.00 ± 0.53	aA	0.93 ± 0.16	aB	0.0 ± 0.0	aC
	Chlorantraniliprole	45^f^	ST	0.07^e^ ± 0.0	bA	0.07 ± 0.0	bA	0.14 ± 0.09	aA
CV (%)	19.56								

^a^Means with the same lower case letter in the same column do not differ significantly (Tukey *P* = 0.01);

^b^Means with the same uppercase letter on the same row do not differ significantly (Tukey *P* = 0.01);

^c^Insecticide applied via foliar spraying;

^d^Insecticide applied via seed treatment;

^e^LOD—Limit of Detection (mg/kg): Chlorantraniliprole: LOD = 0.075;

^f^Milligrams of active ingredient for every 60,000 seeds.

Cyantraniliprole was tested at different dosages (50 and 150 g a.i. ha^-1^) via foliar application in VE and V3, in which the highest dosage of 150 g a.i. ha^-1^ resulted in a significantly high concentration of the active ingredient in leaves V1 (26.94 ± 6.42 mg a.i. kg^-1^) and V4 (71.59 ± 11.72 mg a.i. kg^-1^) (Table 2). However, when applied in stage VE, the insecticide was not detected in leaves of stage V3 (0.0 ± 0.0 mg a.i. kg^-1^) on both rates. When sprayed at V3, cyantraniliprole at 150 g a.i. ha^-1^ was detected on leaves V6 (0.27 ± 0.17 mg a.i. kg^-1^), but did not differ from the foliar spraying at 50 g a.i. ha^-1^ and ST at 150 g a.i. ha^-1^. In application via ST, cyantraniliprole was detected in all the leaves collected and evaluated (V1-V6), which confirms the absorption, translocation, and permanence of the active ingredient in the plant tissues over time.

**Table 2:** Foliar concentration of active ingredient cyantraniliprole applied via foliar spraying and in seed treatment for different leaves of corn plants.

	Treatment	Dosage (g a.i.)	Application type	Leaves (mg a.i. kg^-1^)					
				**V1**		**V2**		**V3**	
**VE X ST**	Cyantraniliprole	50	Foliar ^c^	2.66 ± 0.18	^a^bA	1.73 ± 1.58	aA^b^	0.0 ± 0.0	aA
	Cyantraniliprole	150	Foliar	26.94 ± 6.42	aA	4.74 ± 0.38	aB	0.0 ± 0.0	aB
	Cyantraniliprole	150^f^	ST^d^	6.03 ± 0.76	bA	1.83 ± 0.11	aAB	0.64 ± 0.07	aB
CV (%)	42.13								
				**V4**		**V5**		**V6**	
**V3 X ST**	Cyantraniliprole	50	Foliar	9.02 ± 1.86	bA	0.82 ± 0.02	aA	0.0 ± 0.0	aA
	Cyantraniliprole	150	Foliar	71.59 ± 11.72	aA	2.94 ± 0.35	aB	0.27 ± 0.17	aB
	Cyantraniliprole	150	ST	^e^0.15 ± 0.0	bA	0.15 ± 0.0	aA	0.15 ± 0.0	aA
CV (%)	44.04								

^a^Means with the same lower case letter in the same column do not differ significantly (Tukey *P* = 0.01);

^b^Means with the same uppercase letter on the same row do not differ significantly (Tukey *P* = 0.01);

^c^Insecticide applied via foliar spraying;

^d^Insecticide applied via seed treatment;

^e^LOD—Limit of Detection (mg/kg): Cyantraniliprole: LOD = 0.15;

^f^ Milligrams of active ingredient per 100 kg/seed.

Additionally, we analyzed if the larvae mortality (%) was in accordance with the chemical quantification of active ingredients on leaves (S2 Table). Pearson correlation shows the highest correlation for chlorantraniliprole and cyantraniliprole applied as foliar spray (0.69–0.85) and the lowest correlation when applied as ST (0.51–0.66).

## Discussion

Anthranilic diamides are important insecticides to manage fall armyworm in corn and the understanding of their translocation in plants through different application forms is a field-relevant information. Based on our data of mortality of fall armyworm and the chemical quantification of chlorantraniliprole and cyantraniliprole, it was possible to characterize the translocation of these diamides in corn plant tissues when applied via ST and foliar spraying. It was observed that anthranilic diamides were detected in tissues that did not receive direct application, especially for seed treatment. In this form of application, both insecticides caused larvae mortality > 40% when larvae were feed with corn leaves at V6 stage (Fig 2).

In practice, considering the field-rate of each insecticide, it would represent a long residual effect (± 30 days) of seed treatment, protecting the plants from the damage of *S*. *frugiperda*. The early stages of corn (from VE to V6) are critical for the establishment of plant stand and for the damage of *S*. *frugiperda* on leaves, whorl and stem, where the plants can be completely destroyed [26]. Corn plants expressing Cry proteins require insecticidal sprays to efficient manage *S*. *frugiperda* [5], mainly after V2 stage on the late-planted season (Burtet L., personal information). Therefore, the protection of seed treatment with anthranilic diamides in early stages would reduce or retard a foliar spray to control *S*. *frugiperda* in non-Bt corn or Bt corn events that are ineffective.

The physicochemical features of molecules allowed the absorption of active ingredients by plant roots and the later transportation to all leaves. The entry and translocation of diamides have already been reported in beans [18] and rice [19–27] plants when applied in the sowing groove, seed treatment and foliar spray. The appearance of new leaves and the cycle advancement reduced the mortality of larvae, because of the reduction in concentration of active ingredients in the leaves. However, even at low concentrations, chlorantraniliprole and cyanthraniliprole caused larvae mortality, characterizing their residual effect throughout the evaluations until V6. The long-lasting residual action has already been reported for chlorantraniliprole and cyantraniliprole to control *D*. *platura* and *O*. *nubilalis* [18]. Our results also showed that the insecticide translocation increased the mortality of larvae when applied in seed treatment, even at lower concentrations, in comparison to foliar spraying (Fig 2). The mobility was provided with the entry of insecticides into leaf tissues.

The mobility and translocation capacity, as well as the redistribution of the active ingredient from treated to untreated tissues, is essential to reduce the damage and manage pests that are difficult to control. This includes *S*. *frugiperda*, as it survives inside the corn plant whorl, hindering chemical control by foliar spraying. Anthranilic diamides applied in ST may be an alternative for insect pest management and for the reduction of production costs [18].

However, the amount of active ingredient needed for control varies according to the species and its life stages. *S*. *frugiperda* requires a higher dose of cyantraniliprole than chlorantraniliprole, because LC_50_ of cyantraniliprole for *S*. *frugiperda* is approximately six times greater than for chlorantraniliprole [10]. Therefore, neonate larvae require a lower amount of active ingredient when compared to late larvae stages.

Insecticides applied by foliar spraying were not detected on all leaves evaluated after spraying, meaning that the active ingredients are not mobile via phloem. Chlorantraniliprole proved to be absorbed and translocated upward in soybean plants only when applied to stems or sprayed throughout the plant [7]. This application in the leaves and petioles did not result in the mortality of larvae feeding on new leaves, confirming the hypothesis that chlorantraniliprole is translocated only via xylem [7]. However, our results showed that chlorantraniliprole and cyantraniliprole, when applied in foliar spraying, were not translocated from treated to new untreated leaves of corn plants. Possibly, the presence of leaf sheaths in corn plants hinders the absorption of the active ingredient, preventing it from reaching the vascular system of xylem. Therefore, the insecticide translocation is not only related to physicochemical features of the molecule or environmental conditions, but also associated to plant species and its features at the time of application.

The detection of both insecticides in the leaves that appeared after the application is related to the morphological features of corn plants in growing leaves simultaneously. At both application times (VE and V3), the leaves V1 and V4 were also growing and expanded causing the interception of insecticides at the time of spraying, similar to field applications. Preliminary studies show that when only the third leaf was exposed to insecticides with the rest of the plant covered, the mortality of larvae fed with leaves appearing after application (V4, V5 and V6) was not significant, confirming that the detection of insecticides was not related to ingredient translocation. In addition, the results showed that foliar application and increased dosages did not influence on the mobility of active ingredients in the plant. The absence of translocation of insecticides from treated leaves to new untreated leaves when sprayed via foliar, show that a new application is necessary in a short time to maintain plant protection against new infestation of hatched larvae.

The comprehension about the translocation of active ingredients in corn plants is crucial for choosing the best control tactic on the Integrated Pest Management (IPM) of corn insect pests. Seed treatment proved that both insecticides are mobile only via xylem and the foliar spraying did not show translocation to new leaves. The use of anthranilic diamides in seed treatment offers an important alternative for the management of *S*. *frugiperda* in corn fields, resulting in a long lasting control action, reducing the number of foliar applications of insecticides, as well as controlling costs and reducing the impact on non-target species and in the environment.

## Supporting information

S1 Table Statistical analysis of foliar concentration of chlorantraniliprole and cyantraniliprole applied in foliar spraying and seed treatment for different leaves of corn plants. (DOCX)

S2 Table Pearson correlation between larvae mortality and chemical quantification from V1 to V6 corn stages. (DOCX)

## References

[1] de Boer GJ, Satchivi N. Comparison of translocation properties of insecticides versus herbicides that leads to efficacious control of pests as specifically illustrated by isoclast^™^ active, a new insecticide, and arylex^™^ active, a new herbicide In: Myung K, Satchivi NM, Kingston CK, editors. Retention, Uptake, and Translocation of Agrochemicals in Plants. Washington, DC: American Chemical Society; 2014 p. 75–93.

[2] Cruz I, Figueiredo M, Oliveira AC, Vasconcelos CA. Damage of Spodoptera frugiperda (Smith) in different maize genotypes culti- vated in soil under three levels of aluminium saturation. Int J Pest Manag. 1999;45:293–96.

[3] Carvalho RA, Omoto C, Field LM, Williamson MS, Bass C. Investigating the Molecular Mechanisms of Organophosphate and Pyrethroid Resistance in the Fall Armyworm *Spodoptera frugiperda*. PloS ONE. 2013;8 (4):e62268 10.1371/journal.pone.0062268 23614047 PMC3629120

[4] Bernardi D, Salmeron E, Horikoshi RJ, Bernardi O, Dourado PM, Carvalho RA, et al Cross-Resistance between Cry1 proteins in fall armyworm (Spodoptera frugiperda) may affect the durability of current pyramided Bt maize hybrids in Brazil. PLoS One. 2015;10:e0140130 10.1371/journal.pone.0140130 26473961 PMC4608726

[5] Burtet LM, Bernardi O, Melo AA, Pes MP, Strahl TT, Guedes JVC. Managing fall armyworm, Spodoptera frugiperda (Lepidoptera: Noctuidae), with Bt maize and insecticides in southern Brazil. Pest Manag Sci. 2017;73:2569–77. 10.1002/ps.4660 28695664

[6] Thrash B, Adamczyk JJ, Lorenz JG, Scott AW, Armstron JS, Pfannenstiel R, et al Laboratory evaluations of lepidopteranactive soybean seed treatments on survivorship of fall armyworm (Lepidoptera: Noctuidae) larvae. Flor Entomol. 2013; 96:724–28.

[7] Adams A, Gore J, Catchot A, Musser F, Cook D, Krishnan N, et al Residual and Systemic Efficacy of Chlorantraniliprole and Flubendiamide Against Corn Earworm (Lepidoptera: Noctuidae) in Soybean. J Econ Entomol. 2016;109:2411–17. 10.1093/jee/tow210 27707947 PMC5225962

[8] Jeanguenat A. The story of a new insecticidal chemistry class: the diamides. Pest Manag Sci 2013;69:7–14. 10.1002/ps.3406 23034936

[9] (IRAC) Insecticide Resistance Action Committee. 2017. IRAC mode of action classification scheme. Version 8.2 [Online]. http://www.irac-online.org/documents/moa-classification/ (acesso julho de 2017).

[10] Selby TP, Lahm GP, Stevenson TM. A retrospective look at anthranilic diamide insecticides: discovery and lead optimization to chlorantraniliprole and cyantraniliprole. Pest Manag Sci. 2016;73:658–65. 10.1002/ps.4308 27146435

[11] AGROFIT. Relatório de produtos formulados; 2019; http://agrofit.agricultura.gov.br/agrofit_cons/principal_agrofit_cons.

[12] Lahm GP, Cordova D, Barry JD,.New and Selective ryanodine receptor activators for insect control. Bioorg Med Chem. 2009;17:4127–33. 10.1016/j.bmc.2009.01.018 19186058

[13] FAO. Pesticide residues in food 2013. Joint FAO/WHO Meeting on Pesticide Residues.

[14] Foster SP, Denholm I, Rison Jean-Luc, Portillo HE, Margaritopoulis J, Slater R. Susceptibility of standard clones and European field populations of the green peachaphid, Myzus persicae, and the cotton aphid, Aphis gossypii (Hemiptera:Aphididae), to the novel anthranilic diamide insecticide cyantraniliprole, Pest Manag Sci. 2012; 68:629–33. 10.1002/ps.2306 22045565

[15] Barry JD, Portillo HE, Annan IB, Cameron RA, Clagg DG, Dietrich RF, et al Movement of cyantraniliprole in plants after foliar applications and its impact on the control of sucking and chewing insects. Pest Manag Sci. 2014; 71:395–403 10.1002/ps.3816 24771486

[16] Zhang Y, Lorsbach BA, Castetter S, Lambert WT, Kister J, Wang NX, et al Physicochemical property guidelines for modern agrochemicals. Pest Manag Sci. 2018;74:1979–91. 10.1002/ps.5037 29667318

[17] Tomlin CDS. The Pesticide Manual: A World Compendium. 1457 pages 20° edição. Ed. Hardback.

[18] Schmidt-Jeffris RA, Nault BA. Anthranilic Diamide Insecticides Delivered via Multiple Approaches to Control Vegetable Pests: A Case Study in Snap Bean. J Econ Entom. 2016; 109:1–10. 27760786 10.1093/jee/tow219

[19] Sidhu JK, Hardke JT, Stout MJ. Efficacy of dermacor-x-100^®^ seed treatment against *Diatraea saccharalis* (lepidoptera: crambidae) on rice. Flor Entomologist. 2014;97: 224–32.

[20] Cloyd RA, Bethke JA, Cowles RS. Systemic Insecticides and Their Use in Ornamental Plant Systems. *Flor Ornam Biotech*. 2011; 5:1–9.

[21] Greene GL, Leppla NC, Dickerson WA. Velvetbean Caterpillar: A Rearing Procedure and Artificial Medium. J Econ Entomol. 1976;69:487–88.

[22] Prestes OD, Friggi CA, Adaime MB, Zanella R. QuEChERS—Um método moderno de preparo de amostra para determinação multirresíduo de pesticidas em alimentos por métodos cromatográficos acoplados à espectrometria de massas. Quim Nova. 2009;32:1620–34.

[23] Pihlström T, Fernández-Alba AR, Gamón M, Poulsen ME, Lippold R, Anastassiades. SANTE. Guidance Document on Analytical Quality Control and Method Validation Procedures for Pesticides Residues Analysis in Food and Feed. Safety of the Food Chain Pesticides and biocides. Almeria, Spain: European Commission Document SANTE/11945/2015. (2015)

[24] Abbott WS. A method of computing the effectiveness of an insecticide. J Econ Entomol.1925;18:265–67. 3333059

[25] Ferreira DF. Sisvar: a computer statistical analysis system. Cienc E Agrotecnol. 2011;35:1039–42.

[26] Silva PR, Foresti J. Suscetibilidade do Milho ao Ataque da Lagarta-do-Cartucho. 2016. http://www.pioneersementes.com.br/blog/125/suscetibilidade-do-milho-ao-ataque-da-lagarta-do-cartucho

[27] Chen X, Ren Y, Meng Z, Lu C, Gu H, Zhuang Y. Comparative Uptake of Chlorantraniliprole and Flubendiamide in the Rice Plant. J Agricul Sci. 2015; 12:238–46.

