# Supplementary material for: Translocation of chlorantraniliprole and cyantraniliprole applied to corn as seed treatment and foliar spraying to control Spodoptera frugiperda (Lepidoptera: Noctuidae)
Source: PLoS One. 2020 Apr 1;15(4):e0229151. doi: 10.1371/journal.pone.0229151 (PMC7112192; doi:10.1371/journal.pone.0229151)
Supplement: S1 Table — (DOCX) [file pone.0229151.s001.docx]

S1 Table. Statistical analysis of foliar concentration of chlorantraniliprole and cyantraniliprole applied in foliar spray and seed treatment for different leaves of corn plants.

| **Variable** | ***Df*** | ***F_C_*** | ***P*** |  |
| --- | --- | --- | --- | --- |
|  |  |  |  |  |
| Chlorantraniliprole VE X ST | | | | |
| Application forms | 1 | 15.884 | < 0.01 |  |
| Leaves | 2 | 247.657 | < 0.01 |  |
| Application forms *vs* Leaves | 2 | 16.188 | < 0.01 |  |
| Total | 23 |  |  |  |
| CV (%) | 14.6 |  |  |  |
| Chlorantraniliprole V3 X ST | | | | |
| Application forms | 1 | 530.162 | < 0.01 |  |
| Leaves | 2 | 370.869 | < 0.01 |  |
| Application forms *vs* Leaves | 2 | 381.957 | < 0.01 |  |
| Total | 23 |  |  |  |
| CV (%) | 19.56 |  |  |  |
| Cyantraniliprole VE X ST | | | | |
| Application forms | 2 | 60.587 | < 0.01 |  |
| Leaves | 2 | 94.753 | < 0.01 |  |
| Application forms *vs* Leaves | 4 | 43.649 | < 0.01 |  |
| Total | 35 |  |  |  |
| CV (%) | 44.04 |  |  |  |
| Cyantraniliprole V3 X ST | | | | |
| Application forms | 2 | 137.762 | < 0.01 |  |
| Leaves | 2 | 173.199 | < 0.01 |  |
| Application forms *vs* Leaves | 4 | 122.455 | < 0.01 |  |
| Total | 35 |  |  |  |
| CV (%) | 42.13 |  |  |  |

| \| \| Silva PR, Foresti J. [Suscetibilidade do Milho ao Ataque da Lagarta-do-Cartucho](http://www.pioneersementes.com.br/blog/125/suscetibilidade-do-milho-ao-ataque-da-lagarta-do-cartucho). 2016. \| \| --- \| \| \| --- \| --- \| |
| --- | --- | --- |

<http://www.pioneersementes.com.br/blog/125/suscetibilidade-do-milho-ao-ataque-da-lagarta-do-cartucho>

Chen X, Ren Y, Meng Z, Lu C, Gu H, Zhuang Y. Comparative Uptake of Chlorantraniliprole and Flubendiamide in the Rice Plant. J Agricul Sci. 2015; 12:238-46.
