# Supplementary material for: Translocation of chlorantraniliprole and cyantraniliprole applied to corn as seed treatment and foliar spraying to control Spodoptera frugiperda (Lepidoptera: Noctuidae)
Source: PLoS One. 2020 Apr 1;15(4):e0229151. doi: 10.1371/journal.pone.0229151 (PMC7112192; doi:10.1371/journal.pone.0229151)
Supplement: S2 Table — (DOCX) [file pone.0229151.s002.docx]

**S3 Table. Pearson correlation between larvae mortality and chemical quantification from V1 to V6 corn stages.**

| Correlation of larvae mortality (%) vs active ingredient concentration (mg a.i. kg^-1^) | | | |
| --- | --- | --- | --- |
| Chlorantraniliprole / ST | 0.663789 | Cyantraniliprole / ST | 0.5177966 |
| Chlorantraniliprole / FS | 0.7397482 | Cyantraniliprole 50 / FS | 0.8535574 |
|  |  | Cyantraniliprole 150 / FS | 0.696294 |
